# Effect of crude glycerin levels on meat quality and carcass characteristics of crossbred Boer goats

**DOI:** 10.1002/fsn3.2839

**Published:** 2022-03-24

**Authors:** Higor Fábio Carvalho Bezerra, Edson Mauro Santos, Gleidson Giordano Pinto de Carvalho, Juliana Silva de Oliveira, Anderson de Moura Zanine, Ricardo Martins Araujo Pinho, Maria Leonor Garcia Melo Lopes de Araújo, Alexandre Fernandes Perazzo, Daniele de Jesus Ferreira

**Affiliations:** ^1^ Department of Animal Science Federal University of Paraíba Joao Pessoa Brazil; ^2^ Department of Animal Science Federal University of Bahia Salvador Brazil; ^3^ Department of Animal Science Federal University of Maranhão Chapadinha Brazil

**Keywords:** biodiesel, conformation, co‐product, glycerol, *Longissimus dorsi*, morphometry

## Abstract

This study assessed the effects of four levels of crude glycerin (0, 50, 100, and 150 g/kg dry matter (DM) basis) in the diet of Boer crossbred goat kids on the qualitative and quantitative carcass characteristics as well as meat quality. Thirty‐two crossbred, castrated Boer x undefined breed goat kids with an initial average weight of 17.8 ± 2.2 kg between 3 and 4 months of age were distributed across a complete randomized experiment with four treatments and eight replicates. The DM intake linearly decreased (*p* < .05) as the crude glycerin inclusion level in the diet increased. Crude glycerin levels decreased (linear effect, *p* < .05) empty body weight, hot dressing percentage, and cold dressing percentage. Conformation and subcutaneous fat thickness were not affected (*p* > .05) by dietary crude glycerin. Crude glycerin levels decreased (linear effect, *p* = .03) rib eye area of the *Longissimus dorsi* muscle, however, did not affect color, cooking loss, and shear force. The crude glycerin can be included up to 50 g/kg DM in the diet of crossbred Boer goats without negatively affecting carcass characteristics and meat quality. It can be recommended as an energy source in finishing diets.

## 1 INTRODUCTION

With periodically increasing feed costs, alternative food sources such as crude glycerin (a glycerol‐rich source) have become more prominent (Chanjula et al., 2015; Semkiv et al., 2020). The use of crude glycerin avoids its disposal in the environment and contributes to the energetic demands of animals capable of converting this co‐product into foods of high nutritional value for humans while also replacing ingredients in animal feed (e.g., corn) whose price varies greatly throughout the year.

The glycerol present in the animal body is usually linked to a lipid source (triglycerides, lipoproteins, or dietary fats). When used in animal feed, however, glycerol is not linked to such sources and is assimilated as an energy source primarily as glucose (Bergman et al., 1968; Carvalho et al., 2015). Only when consumed in excess will glycerol be converted into glycogen in the liver and later transform into fat through lipogenesis. Possibly glycerin does not cause harmful changes to the meat of animals that consume it. Nevertheless, the use of crude glycerin in the diet of ruminants leads to a high availability of gluconeogenic components because of the conversion of glycerin into propionate, which can be used in fatty acid synthesis (Versemann et al., 2008) and affect meat quality.

Thus, this study assessed the effects of including levels of crude glycerin in the diet of Boer crossbred goat kids on the qualitative and quantitative carcass characteristics as well as meat quality.

## 2 MATERIALS AND METHODS

### 2.1 Local conditions and period of the experiment

This experiment was conducted at the Experimental Farm of the School of Veterinary Medicine and Animal Science Federal University of Bahia between November 2013 and January 2014. All animal management and care procedures were in accordance with the guidelines approved by Federal University of Bahia Use and Care Committee (n. 08/2013).

### 2.2 Location, animals, and diets

Thirty‐two male Boer crossbred goat kids with an initial average weight of 17.8 ± 2.2 kg and age ranging from 3 to 4 months were allocated in a completely randomized block design with four levels of crude glycerin (0, 50, 100, and 150 g/kg of (dry matter [DM] basis) and eight replicates (Table 1). Two weeks before starting the study, all animals were vaccinated and dewormed for ecto‐ and endoparasites and received sorghum silage as roughage ad libitum and increasing proportions of the experimental feeds. Afterwards, they were housed in individual pens within a covered shed with slatted, suspended floors, and equipped with drinking fountains and feeding troughs to ensure ad libitum access to water and food.

**TABLE 1:** Participation of ingredients and the chemical composition of the experimental diets

Ingredient	Dietary crude glycerin level (g/kg of DM)			
	0	50	100	150
Dietary ingredient (g/kg of DM)				
Corn meal	180.00	120.00	60.00	0.00
Soybean meal	205.00	215.00	225.00	235.00
Crude glycerin	0.00	50.00	100.00	150.00
Mineral supplement^a^	15.00	15.00	15.00	15.00
Sorghum silage	600.00	600.00	600.00	600.00
Chemical composition (g/kg of DM)				
Dry matter	554.60	557.20	559.80	562.30
Organic matter	941.00	937.10	940.80	941.80
Mineral matter	50.80	52.30	53.90	55.40
Crude protein	149.20	149.80	150.50	151.10
Ether extract	31.30	28.40	25.50	22.60
Neutral detergent fiber	349.40	343.10	336.80	330.50
Acid detergent fiber	166.70	166.30	165.90	165.50
Total digestible nutrients	639.80	639.90	639.90	640.00
Methanol	0.00	3.30	6.60	9.90

^a^ Mineral supplement (nutrient/kg supplement): vitamin A, 135,000 IU; vitamin D3, 68,000 IU; vitamin E, 450 IU; calcium, 240 g; phosphorus, 71 g; potassium, 28.2 g; sulfur, 20 g plus sulfur from ammonium sulfate to maintain the 9:1 ratio (urea:ammonium sulfate); magnesium, 20 g; copper, 400 mg; cobalt, 30 mg; chromium, 10 mg; iron, 2500 mg; iodine, 40 mg; manganese, 1350 mg; selenium, 15 mg; zinc, 1700 mg; maximum fluorine, 710 mg; phosphorus solubility in 2% citric acid (minimum).

The goats were fed twice a day and in two equal portions, at 8 a.m. and at 4 p.m. The feed was individually weighed in a roughage:concentrate ratio of 60:40 and subsequently mixed to minimize selectiveness by the animals. Diets were formulated to be isonitrogenous (150 g/kg of CP) and isoenergetic (640 g/kg of TDN) and to meet the nutritional requirements for goats with an estimated potential average weight gain of 150 g/day (NRC, 2007).

Chemical composition was determined according to AOAC (1990). Dry Matter (method 934.01), Mineral matter (method 930.05), Crude protein (method 920.87), and Ether extract (method 920.85). Determination of the acid detergent fiber (ADF) and neutral detergent fiber (NDF) was obtained following a published method (Van Soest, 1991), the values of total digestible nutrients (TDN) were determined according to the method described by Mertens (2002) = Value expressed in % of DM =TDN (%) = DCP +DNFC + (DEE ×2.25) + DNDF, where DCP is the digestible CP, DEE is the digestible EE, DNDF is the digestible NDF, and DNFC is digestible NFC, performed at the Laboratory of Animal Nutrition of the Federal University of Bahia.

### 2.3 Carcass and meat characteristics

The experiment lasted 3 months. All goats were fasted (drinking water was no withdrawn) for 16 h before recording the final live weight (LW).

The lambs were slaughtered at a commercial abattoir and the following data were recorded: body weight at slaughter (BWS), empty body weight (EBW), hot carcass weight (HCW), cold carcass weight (CCW), hot dressing percentage (HDP), and cold dressing percentage (CDP). The CCW and CDP were determined after 24‐h cooling period (4°C for 24 h).

After weighing, the following carcass morphological measures were examined following Cézar and Souza (2007): the distance between the cervicothoracic joint and the first intercoccygeal joint (external carcass length, ECL); the distance between anterior edge of pubic bone and anterior edge of first rib at its midpoint (internal carcass length, ICL); All parameter measurements were taken using a metric measuring tape.

The left half‐carcasses were cross‐sectioned between the 12th and 13th thoracic vertebra, where the subcutaneous fat thickness (SFT) on the *longissimus muscle* was determined with a digital caliper. The conformation and SFT of the *longissimus muscle* were determined in a 1–5 scale according Osorio and Osorio (2005). In addition, measurements were taken of the maximum width (A) and the maximum depth (B). The rib eye area was assessed using the formula: REA = (A/2 × B/2) × π.

Meat color was evaluated using a Minolta CR300 colorimeter (Minolta,1998) operating in the CIE (L*, a*, b*) system, in which L* represented lightness, a*, the intensity of the color red, and b*, the intensity of the color yellow (Cañeque & Sañudo, 2001; Miltenburg et al., 1992).

Cooking loss (CL) was determined in each loin sample with approximately1.5 cm thickness, 3.0 cm length, and 2.5 cm width (Duckett et al., 1998). The meat texture was determined by the shear force (SF), by adopting the method described by Wheeler et al. (1995), expressed in kgf.

### 2.4 Statistical analysis

Data were prepared using analysis of variance. The initial weight of goats was considered a covariate in the statistical model. The results were interpreted through decomposition of the orthogonal polynomials in linear and quadratic using the PROC MIXED function of the SAS (2003) software (version 9.1).

The homogeneity of variance between treatments was assumed, and the degrees of freedom were estimated using the Kenward–Roger method. The regression models were adjusted according to the significance of the parameters β1 and β2 by using the method of restricted maximum likelihood in PROC MIXED, and the estimation of parameters was obtained through the PROC REG function of the SAS software (version 9.1). All statistical procedures were performed using the value of 0.05 as the critical level of probability for error type I.

## 3 RESULTS

### 3.1 Carcass characteristics

The DM intake linearly decreased (*p* < .05) as the crude glycerin inclusion level in the diet increased. Crude glycerin levels decreased (linear effect, *p* < .05) empty body weight (EBW), hot carcass weight (HCW), and cold carcass weight (CCW) (Table 2).

**TABLE 2:** Carcass characteristics of Boer crossbred goats fed diets containing crude glycerin

Variable	Crude glycerin inclusion level (%)				*SEM*	*p*‐value	
	0	5	10	15		L^†^	Q^‡^
Intake DM (g/day)	827	733	714	608	27.4	.01	.89
BWS (kg)	26.17	25.15	24.90	23.09	0.6047	.0404	.7460
EBW (kg)	22.17	21.60	20.12	17.76	0.8879	.0387	.6014
HCW (kg)	11.27	10.18	9.94	9.24	0.2852	.0173	.6824
CCW (kg)	11.22	10.12	9.87	9.19	0.2888	.0153	.7044
CL (%)	0.48	0.50	0.75	1.10	0.1299	.0597	.5029
HDP (%)	43.12	40.59	39.94	40.24	0.6102	.1267	.2432
CDP (%)	42.92	40.38	39.66	39.80	0.6193	.0971	.2738

	Regression equations	
Intake DM	Ŷ = 821,7073–13,5107X	*r*² = .95
BWS (kg)	Ŷ = 26.2566 – 0.1921X	*r*² = .91
EBW (kg)	Ŷ = 22.5892 – 0.3015X	*r*² = .93
HCW (kg)	Ŷ = 11.0553 – 0.1204X	*r*² = .94
CCW (kg)	Ŷ = 11.0148 – 0.1240X	*r*² = .94

Abbreviations: ^†^, Linear effect; ^‡^, Quadratic effect; BWS, body weight at slaughter; CCW, cold carcass weight; CDP, cold dressing percentage; CL, cooling loss; EBW, empty body weight; HCW, hot carcass weight; HDP, hot dressing percentage; *p*‐value, Significant probability at the 5% level; *SEM*, Standard error of the mean.

The inclusion of crude glycerin did not affect (*p* > .05) cooling loss (CL), hot dressing percentage (HDP), and cold dressing percentage (CDP) (Table 2). The mean CL value was 0.71%, which is lower than previously published results.

### 3.2 Carcass measurements

Conformation and subcutaneous fat thickness (SFT) were not affected (*p* > .05; Table 3) by dietary crude glycerin.

**TABLE 3:** Carcass measurements of Boer crossbred goats fed diets containing crude glycerin

Items	Dietary glycerin level (%)				*p*‐value	*SEM*	*SD*
	0	5	10	15			
Conformation	1.96	2.09	2.04	1.94	.23	0.00	0.35
SFT	1.04	0.94	1.14	1.19	.92	0.00	0.45
ECL (cm)	51.67	51.50	52.86	51.00	.86	0.05	3.29
ICL (cm)	55.83	54.13	55.71	54.48	.12	0.04	2.57

Note

Conformation (1, Very poor; 1.5, Poor; 2, Acceptable; 2.5, Average; 3, Good; 3.5, Very good; 4, Superior; 4.5, Very superior; 5, Excellent); SFT =subcutaneous fat thickness (1, Excessively lean; 1.5, Very lean; 2, Lean; 2.5, Slightly lean; 3, Normal; 3.5, Slightly fatty; 4, Fatty; 4.5, Very fatty; 5, Excessively fatty).

Abbreviations: ECL, external carcass length; ICL, internal carcass length; ns, Not significant (*p* > .05); *p*‐value, Significant probability at the 5% level; *SD*, Standard deviation; *SEM*, Standard error of the mean.

In addition, external carcass length (ECL) and internal carcass length (ICL) were also not influenced (*p* > .05) by crude glycerin levels in the diet (Table 3). Morphometric characteristics were also not significantly (*p* > .05) influenced by diets having the same nutritional composition.

### 3.3 Qualitative characteristics muscle

Crude glycerin levels decreased (linear effect, *p* =.0302) rib eye area (REA) of the *Longissimus dorsi* muscle (Table 4). The SFT of the *Longissimus dorsi* muscle of goats was not affected (*p* > .05).

**TABLE 4:** Qualitative characteristics of the *Longissimus dorsi* muscle of Boer crossbred goats fed diets containing crude glycerin

Items	Dietary glycerin level (%)				*SEM*	*p*‐value	
	0	5	10	15		L^†^	Q^‡^
REA (cm²)	8.17	7.69	7.35	6.55	0.27	.03	.75
SFT (mm)	1.32	1.50	1.40	1.21	0.00	.62	.36
Color component							
L*	36.50	39.66	38.95	39.72	0.56	.07	.28
a*	10.74	11.74	11.55	12.02	0.30	.20	.67
b*	4.99	5.90	6.09	6.33	0.26	.08	.53
CL (%)	29.97	35.00	30.07	35.86	1.19	.22	.87
SF (kgf)	1.97	3.17	2.36	2.92	0.20	.26	.43

	Regression equations			
REA (cm²)	Ŷ = 8.2279 – 0.1055X		*r*² = .97	

Abbreviations: ^†^, Linear effect; ^‡^, Quadratic effect; CL, Cooking loss; L*, lightness, a*, redness, b*, yellowness; *p*‐value, Significant probability at the 5% level; REA, Rib eye area; *SEM*, Standard error of the mean; SF, Shear force; SFT, Subcutaneous fat thickness.

Glycerin levels did not affect (*p* > .05) the physical goat meat characteristics (Table 4). The cooking loss (CL) did not differ (*p* > .05) among crude glycerin levels, showing loss values ranging from 29.97% to 35.86%. The different levels of crude glycerin did not influence shear force (SF) (*p* > .05).

## 4 DISCUSSION

Crude glycerin levels decreased EBW, HCW, and CCW. These characteristics were expected to be negatively affected after including increasing levels of crude glycerin because the resulting decreased feed intake should have contributed to a decrease in weight gain and therefore similar EBW, HCW, and CCW. Lage et al. (2014) described similar results in their study of the effects of increasing levels of crude glycerin (30, 60, 90, and 120 g/kg) in the diets of feedlot sheep based on carcass weight.

In their evaluation of carcass traits of undefined breed (UDB) x Anglo Nubian and UDB x Boer crossbred goat kids slaughtered at different body weights, Oliveira et al. (2008) did not find differences between weights or between breeds, finding an average of CL of 2.01, which might indicate that the use of glycerin in goat termination does not promote CL increases. We found that cooling was duly performed and that this effect imbued a characteristic required by slaughterhouses to improve CDP and increase profits as well as increase meat value by avoiding excessive carcass water loss and preventing meat toughness.

Crude glycerin did not affect HDP and CDP most likely because these variables are influenced by factors such as breed, age, slaughter weight, sex, and breeding system. Furthermore, factors such as feeding show little influence on these variables except when diet composition relative to amount of nutrients varies greatly. As such, these variables were not expected to change because the animals were part of a homogeneous lot and their diets were balanced.

Conformation and subcutaneous fat thickness were not affected by dietary crude glycerin. According to Lisboa et al. (2010), these variations are influenced by factors such as breed, genotype, age, sex, and breeding system. Dietary changes do not strongly influence these characteristics, provided that nutrient contents remain similar. However, when the amounts of nutrients such as energy source and protein are changed, these variables can be influenced (Cartaxo et al., 2011). In their assessment of the effect of two energy levels (2.4 and 2.9 Mcal metabolizable energy [ME]/kg DM) and different lamb genotypes (Santa Inês, Santa Inês x Dorper, and Santa Inês x UDB) on carcass characteristics, the authors observed both genotype and dietary energy‐level effects in which animals receiving diets with higher energy levels showed better carcass conformation and SFT.

The ECL and ICL were also not influenced by crude glycerin levels in the diet. This result is similar to that described by Gunn et al. (2010) and Barros et al. (2015), which studied the inclusion of crude glycerin levels (0, 15, 30, and 45%) in the diet of feedlot sheep. Morphometric characteristics were also not influenced by diets having the same nutritional composition. These results demonstrate that crude glycerin can be used in the diets of finishing Boer crossbred goats without any depreciation to goat carcasses.

The decrease in the loin eye area (LEA) of the *Longissimus dorsi* muscle can be explained by the lower weight gains associated with increasing glycerin levels that negatively influenced the fat and muscle deposition of these animals. Fat deposition in animal carcasses is related to aspects such as breed, age, sex, and breeding system, and the increased REA is due to the greater BWS and age. As such, this objective measure enables the prediction of the amount of muscle in the carcass (Silva Sobrinho et al., 2008).

The SFT of the *Longissimus dorsi* muscle of goats was not influenced, which can be explained by the small amount of subcutaneous fat in goats, given that the largest fat deposition in these animals occurs in the abdominal and thoracic cavities.

According to Khliji et al. (2010), the acceptable limit for L* in lamb is 34–35, which is lower than the values found in this study. Meat color is a relevant characteristic at the time of purchase because it is the primary criterion used by consumers when selecting a product (except when undesired odors are present). Values obtained in this study are in agreement with those mentioned by Chanjula et al. (2015).

Cooling losses are related to those that occur during the meat preparation or cooking process and are associated with yield regarding the preparation for consumption; moreover, they influence the meat's juiciness. The shear force (SF) presented values between 1.97 and 3.17, which is classified as "soft" based on Cézar and Souza (2007). Shear force is used to measure meat tenderness, and higher SF values denote lower meat tenderness. The values observed in this study were below with those mentioned by Lemes et al. (2013), which ranged from 3.0 to 4.7 kgf in Angorá, Crioulo, and Zebu goats (Anglo Nubian crossbreeds). The values found by the current study demonstrate that meats show great tenderness when not affected by crude glycerin.

## 5 CONCLUSIONS

The crude glycerin can be included up to 50 g/kg DM in the diet of crossbred Boer goats without negatively affecting carcass characteristics and meat quality. It can be recommended as an energy source in finishing diets.

## CONFLICT OF INTEREST

The author has no conflict of interest to declare.

## Data Availability

Os dados que suportam os resultados deste estudo estão disponíveis abertamente em [nome do repositório, por exemplo, “figshare”] em http://doi.org/[doi], número de referência [número de referência].
